# Factors Associated With Satisfaction and Perceived Change Among University Students After Implementing Care Practices to Address Psychological Distress

**DOI:** 10.1155/nrp/5549256

**Published:** 2026-11-14

**Authors:** Tatiane Carolina Martins Machado Rodrigues, Guilherme Correa Barbosa

**Affiliations:** ^1^ Nursing Department Medical School São Paulo State University Botucatu Campus, Botucatu São Paulo Brazil unesp.br

**Keywords:** coping strategies, health assessment, student psychological distress, student satisfaction

## Abstract

**Introduction:**

Entering university symbolizes a sudden transition for most students, who are faced with an unfamiliar environment, new demands, and adaptations associated with individual and collective expectations regarding entry into higher education, which may be linked to the onset of psychological distress.

**Objective:**

To analyze the factors associated with the repercussions of satisfaction and perceived change among university students after implementing care practices to address psychological distress.

**Method:**

A cross‐sectional study involving 540 students from a public federal university in Brazil. The assessment was conducted using the Patient Satisfaction with Mental Health Services Scale and the Perceived Change Scale. The analysis included descriptive statistics and multiple linear regression models, with *p* < 0.05 set as significance level.

**Results:**

Higher scores for satisfaction and perceived change were associated with participation in activities such as Circle Dance and psychological counseling, as well as with sense of belonging to the campus. In contrast, students who self‐identified as Indigenous, of “Other” gender, in their fourth year of study and those who required housing assistance and social services had lower mean scores on the scales.

**Conclusion:**

Participating in care practices promotes well‐being and adaptation. However, initiatives must account for the pre‐existing vulnerability of minority and socioeconomically disadvantaged groups, whose continued enrollment requires ongoing institutional support.

**Summary**
• What is known about the subject matter?
◦ University life is marked by a time of renewing individual and collective experiences that require discipline, new responsibilities, and developing academic skills that can exert negative impacts on academic performance and contribute to psychological distress.
• What does the article add to our knowledge?
◦ It is important to understand what is causing distress in students, especially in order to prevent it, with the intention of promoting measures that seek to reduce existing inequalities in this population segment through actions that promote health and well‐being.
◦ Along with the interprofessional work of health and student support teams, institutional efforts to encourage partnerships and theoretical frameworks both within and outside the university have the potential to ease higher education experiences characterized by positive outcomes, mitigating risk factors for illness and promoting mental health.
◦ In charge of trained professionals with the goal of promptly addressing the students’ needs, skilled listening has shown to improve well‐being.
◦ The student support service should be widely publicized, with the aim of improving access to information through the institution’s websites, with specific, clear, and easy‐to‐understand links containing concise and objective content, in order to avoid obstacles to using the service.
• Which are the implications for the practice?
◦ Implementing and assessing activities are important in encouraging actions that truly promote a stigma‐free environment, engaging and welcoming, alleviate psychological distress, and promote the use of strategies that provide pleasure, emphasizing well‐being.
• Statement of relevance
◦ Resulting from a professional PhD thesis in Nursing, the article entitled “Factors associated with satisfaction and perceived change among university students after implementing care practices to address psychological distress” has the potential to exert positive impacts on the clinical practice. Its completion promoted awareness of university mental health issues and strengthened student‐centered care actions. Furthermore, it highlighted the role of Nursing in promoting mental health, implementing prevention and intervention strategies and advocating for public policies that contribute to improving community well‐being and to strengthening the health system.

## 1. Introduction

The mental health challenges faced by humanity have become a constant and pressing concern in the Public Health field, with a recurrent increase in psychological distress and demanding urgent attention to its genesis [1] to be properly addressed, prioritizing a person‐centered focus on continuous and accessible care [2].

In this context, university students constitute a group that is vulnerable to psychological distress, especially in the face of the changes, new demands, and expectations associated with the transition to higher education [3, 4].

This transition has shown to be linked to feelings of insecurity and has been associated with changes in a wide range of factors, including emotional, physical, cognitive, social, and occupational aspects. As a result, students may experience certain decline in their overall health and well‐being [5].

In light of these considerations, teaching institutions are faced with the imperative of responding to multifaceted demands, fostering welcoming environments and health‐promoting practices grounded in diverse care modalities, prioritizing well‐being, and mitigating distress [5, 6].

These practices have been identified as essential tools for enhancing adaptation to the academic environment, increasing confidence in professional training, reducing strain, and promoting health in this population group [6].

In this regard, the World Health Organization (WHO) [7] emphasizes the importance of evaluating the quality and effectiveness of mental health services. However, there are still few studies investigating university students’ satisfaction and perceptions regarding change following their participation in institutional initiatives aimed at addressing psychological distress.

Therefore, with the objective of identifying factors associated with the repercussions of satisfaction and perceived change among university students, this study was conducted using the Brazilian Mental Health Services Satisfaction Scale (SATIS‐BR) and the Perceived Change Scale (PCS). These tools allow assessing treatment outcomes from the students’ own perspective, thereby demonstrating the impact of interventions on their lives [8]. Their use in an educational context is justified by the still limited scientific literature on the subject matter and the scarcity of validated instruments for this population segment [9]. According to the author, education is one of the main domains of students’ lives, so that experiences of satisfaction and perceptions regarding change in this environment can exert a significant impact on their academic and personal paths.

## 2. Objective

This study aims at analyzing the factors associated with the repercussions of satisfaction and perceived change among university students after implementing care practices to address psychological distress. The questions initially asked were as follows: Which sociodemographic and academic factors are associated with satisfaction regarding the services offered? and How do different types of interventions (psychological support, integrative practices, and socioeconomic assistance) relate to the students’ perceptions regarding changes in their quality of life? The central hypothesis of this study is that participating in direct mental health interventions and integrative practices (such as psychological counseling and Circle Dance) is positively associated with satisfaction and change scores. Conversely, it is hypothesized that the need for basic social assistance services is associated with lower scores, reflecting the greater structural vulnerability and pre‐existing baseline distress presented by these groups.

## 3. Materials and Methods

### 3.1. Type of Study

A cross‐sectional associative study aimed at analyzing the outcomes of care practices was developed to address psychological distress in college students, using data collected at a single point in time. This article is part of the first author’s professional PhD dissertation in Nursing.

The theoretical and methodological framework proposed was that of the three‐dimensional assessment of mental health services [10], which allows assessing satisfaction and perceived change among students served after implementing care practices through an assessment model that encompasses the existence of an interrelationship among structure, process, and outcome. This study explores the “outcome” dimension to analyze university students’ perceptions.

### 3.2. Methodological Rigor

The EQUATOR NETWORK Strengthening the Reporting of Observational Studies in Epidemiology (STROBE) instrument was used as data organization criterion [11].

### 3.3. Research Environment

The research was conducted across the four campuses of a federal public university in the state of São Paulo, Brazil. As of May 2023, the university had a total of 23,721 enrolled students and 2321 public employees distributed across its four campuses.

The sector responsible for managing actions and strategies aimed at promoting quality of life and retention among all individuals who constitute this university community is the Dean’s Office for Community and Student Affairs (Pró‐Reitoria de Assuntos Comunitários e Estudantis, ProACE), which oversees the student health and assistance departments.

The purpose of the health departments is to promote and actively participate in primary care actions aimed at disease prevention and health promotion, as well as providing medical, dental, psychological, and nursing care. The campuses also have social workers and administrative staff who provide student assistance services.

### 3.4. Research Participants

Students enrolled at the university and participating in activities offered by ProACE.

### 3.5. Ethical Procedures

The study was approved by the Research Ethics Committee of the sponsoring institution (approval opinion No. 5,766,479; CAAE: 64343022.4.0000.5411; November 21st, 2022). The research methodology was implemented in accordance with Resolution No. 466/12, and all participants provided their informed consent in writing by signing a Free and Informed Consent Form (FICF) [12].

The authors declare no conflicts of interest.

### 3.6. Sampling

The number of participating students was calculated to ensure adequate statistical power for fitting multiple linear regression models, considering up to 5 predictor variables. Adopting a 5% significance level and a minimum statistical power of 80%, and anticipating a 10% dropout rate, the final sample of 528 students was more than sufficient to detect small‐ to medium‐sized effect sizes on the continuous scores obtained in the SATIS‐BR and PCS. Given the population size, the sample was stratified into 308 participants at Campus A, 132 at Campus B, 44 at Campus C, and 44 at Campus D.

### 3.7. Data Collection and Analysis

The interviews with the participating students were conducted between March and December 2024 using a semistructured interview format. Data collection took place in person, following participation in at least one activity or service offered, or via email for those who had also taken part in at least one activity or service provided by the ProACE departments.

The analysis of factors associated with the scores obtained in SATIS‐BR and in the overall PCS was performed by fitting multiple linear regression models with a normal response. The variables were retained in their original continuous form. The deterministic component of the model included variables that showed statistical associations with *p* < 0.20 in the bivariate analysis. In the final multiple models, associations were considered statistically significant when *p* < 0.05. The analyses were performed using the Statistical Package for the Social Sciences (SPSS), Version 21.

### 3.8. Data Collection Instruments

The data collection process was guided by a script subdivided into three sections containing sociodemographic information about the participants, the Patient Satisfaction with Mental Health Services Scale (SATIS‐BR) and the PCS.

The sociodemographic questionnaire included the following variables: name (optional), Academic Registration Number (ARA) (optional), date of birth, age, place of birth (city/state), gender identity (cisgender man, transgender man, cisgender woman, transgender woman, other), academic program, year of study, program progression lag in relation to the expected year of study, race/ethnicity (White, Brown, Asian, and Indigenous), campus (A, B, C, and D), having been granted any type of scholarship or financial aid, living with parents/family members, health conditions, name of the health conditions, medication use, name of the medications, follow‐up with a psychologist/therapist/psychiatrist, and duration of follow‐up.

To analyze the practices developed to address psychological distress in college students, the Patient Satisfaction with Mental Health Services Scale (SATIS‐BR) and the PCS were used, both developed by the WHO as part of the WHO‐SATIS project, to serve as tools for assessing mental health services [13] [13].

The SATIS‐BR Satisfaction Scale is part of a set of three scales for assessing mental health services: the User and Family Satisfaction Assessment Scales with Mental Health Services–SATIS‐BR [14, 15] and the Professional Satisfaction Assessment Scale in Mental Health Services–SATIS‐BR [15].

These three scales were adapted and validated in Brazil by Bandeira et al. between 2000 and 2002 and updated in 2012 [14, 15].

SATIS‐BR has 13 items arranged on a 5‐point Likert scale, where 1 represents that the user is very dissatisfied and 5 that they are very satisfied, in addition to a question with 3 items about the service: whether it could be improved, (1) Yes, (2) No, (3) I don’t know, as well as an open space about what might be improved [14].

The model’s three‐factor structure, with eigenvalues greater than 1.0, accounted for 53.12% of the data variance, with all items presenting factor loadings above 0.45. The three factors (or subscales) identified were as follows: (1) team competence and understanding, (2) help and welcoming care, and (3) physical conditions of the service. The first factor consists of seven items (2, 3, 4, 5, 6, 9, and 10), responsible for 25.05% of the data variance and assesses the professionals’ competence and understanding regarding the patients’ problems. The second factor has three items (1, 7, and 8), which explain 14.16% of the data variance and assess the assistance provided to the patients by the service and the quality of the professionals’ welcoming care. The third factor includes two items (11 and 12), which explain 13.92% of the data variance and assess the physical conditions and comfort of the service [14].

Reliability of the SATIS‐BR scale was assessed through an analysis of its internal consistency, calculating Cronbach’s alpha coefficient and demonstrating adequate psychometric properties, internal consistency, and construct validity [14].

Originally developed by Mercier et al. [16] in Canadá, adapted for Brazil by Bandeira et al. [17] and validated by the same authors in 2011, PCS is a scale that assesses treatment outcomes in mental health services in terms of changes perceived by the patients themselves in various dimensions of their lives, showing high internal consistency (*α* = 0.84) [8].

The structural characteristics, the dimensions assessed, the score ranges, and the theoretical cutoff points for interpreting the scores on both instruments are summarized in Table 1.

**TABLE 1:** Summary of the characteristics and scoring parameters of the SATIS‐BR and PCS.

Instrument	Dimensions assessed	Number of items	Score range	Theoretical cutoff point and interpretation
SATIS‐BR (Patient Satisfaction Scale)	1. Team competence and understanding 2. Help and welcoming care 3. Physical conditions of the service	13	5‐Point Likert Scale (From 1 = *Very dissatisfied* to 5 = *Very satisfied*)	3.0 (Neutral) Scores > 3.0: Satisfaction Scores < 3.0: Dissatisfaction

PCS (Perceived Change Scale)	Effects of treatments in general and on various aspects of daily life	19	3‐ Point Likert Scale (1 = *Worse than before*; 2 = *No change*; 3 = *Better than before*)	2.0 (No change) Scores > 2.0: Perceived improvement Scores < 2.0: Perceived worsening

The characteristics of the sample were described using absolute and relative frequencies (percentages) for the categorical variables and mean values and standard deviations (SD) for the quantitative ones. The scores obtained in the Patient Satisfaction with Mental Health Services Scale (SATIS‐BR) and the PCS were used to investigate the factors associated with the outcomes of interest, according to the criteria previously established for each instrument.

Initially, a univariate analysis was performed to examine the association between the students’ sociodemographic/academic characteristics and the categorized satisfaction (SATIS‐BR) and perceived change (PCS) outcomes. Variables with *p* values ≤ 0.20 in the univariate analysis were selected for inclusion in the multivariate model. The decision to use a *p* value threshold of ≤ 0.20 at this stage was intended to provide greater sensitivity in selecting variables potentially associated with the outcomes, thereby avoiding prematurely excluding possible confounding variables or those with explanatory relevance.

In the multivariate analysis, the variables selected were analyzed simultaneously, allowing controlling potential confounding factors and estimating the independent association between sociodemographic/academic characteristics and the outcomes under study. Variables with *p* ≤ 0.05 (the statistical significance level adopted) were retained in the final model. Thus, the multivariate analysis made it possible to identify which characteristics remained associated with satisfaction and perceived change after adjusting for the other variables included in the model. The statistical analyses were performed using the SPSS, Version 21.0.

Regarding convergent validity of the PCS tool, a significant positive Pearson’s correlation was obtained with SATIS‐BR, confirming the postulated hypothesis of a theoretical relationship between the “perceived change” and “satisfaction with the service” constructs [8].

## 4. Results

This study included students enrolled at any of the university’s four campuses and who participated in some activity/service offered by the ProACE departments. Of these, 540 individuals completed the form, either online or in an interview, representing 102% of the planned sample of 528 students, reflecting associations, not effects.

Regarding the study participants’ sociodemographic data, it was observed that 519 (96%) students were enrolled in undergraduate programs; the age group with the highest incidence was 21–24 years old with 228 (42%) students, followed by 18–20 years old with 178 (33%); cisgender females were the most common with 289 (53.5%); 226 (41.9%) were in their first year of the program; the predominant race was white with 320 (59.3%), followed by brown with 109 (20.2%); 326 (60.4%) received some form of financial aid from the university; 421 (78%) did not live with their parents and/or family members; regarding the year they should be studying, 232 (43%) were behind; 176 (33%) reported having some health problem, 210 (39%) were under the care of a psychologist/therapist/psychiatrist; and 179 (67%) took some type of medication.

A wide range of initiatives was made available to the students, encompassing both individual and group activities. The individual activities included welcoming care and qualified listening, early syphilis, hepatitis and HIV diagnosing, guidance on physical and mental health, home visits, psychological support services, emergency psychological care, psychotherapy, medical consultations, nursing consultations, oral health promotion, and guidance on scholarships and student benefits. In turn, the group activities included support groups and roundtable discussions addressing topics such as self‐regulation techniques (mindfulness), Circle Dance, sexual and reproductive health, prevention of violence against university students, prevention of psychological distress (self‐care), welcoming care for first‐year students, guidance on use of alcohol and other psychoactive substances, postsuicide intervention, and transition out of the university.

Regarding users’ satisfaction with the mental health service, the answers were concentrated at scores 4 and 5, reaching the highest frequencies for items one and six at 49.4% and 51.3%, respectively. These findings indicate that approximately 50% of the students analyzed felt respected and did not experience difficulties obtaining information from the healthcare teams (Table 2).

**TABLE 2:** Patient Satisfaction with Mental Health Services Scale (SATIS‐BR).

SATIS	1	%	2	%	3	%	4	%	5	%
1	13	2.4	15	2.8	48	8.9	197	36.5	267	49.4
2	3	0.6	5	0.9	82	15.2	254	47	196	36.3
3	2	0.4	25	4.6	63	11.7	279	51.5	172	31.9
4	3	0.6	28	5.2	71	13.1	262	48.5	176	32.6
5	2	0.4	8	1.5	39	7.2	304	56.3	187	34.6
6	9	1.7	35	6.5	97	18	122	22.3	277	51.3
7	4	0.7	19	3.5	88	16.3	301	55.7	128	23.7
8	1	0.2	16	3	85	15.7	272	50.4	166	30.7
9	1	0.2	9	1.7	52	9.6	307	56.9	171	31.7
10	2	0.4	6	1.1	58	10.7	306	56.7	167	30.9
11	4	0.7	4	0.7	37	6.9	298	55.5	197	36.5
12	4	0.7	15	2.8	74	13.7	317	58.7	130	24.1
13	8	1.5	29	5.4	132	24.4	286	53	85	15.7

It can be seen that the overall prevalence for satisfaction was 83%, with a mean overall Patient Satisfaction Score of 54 (SD = 7.3), ranging from 50.0 to 59.0 within a possible range of 28–65. The results obtained in the subscales showed mean values of 29 (SD = 4.2), 12.3 (SD = 1.9), and 8.2 (SD = 1.2) for the “team competence and understanding,” “help and welcoming care” and “physical conditions of the service” dimensions, respectively (Table 3).

**TABLE 3:** Assessment of satisfaction levels among the university students who took part in the activities offered by ProACE.

Satisfaction level (*n* = 540)	Mean ± SD	Minimum	Maximum
Global	54 ± 7.3	50	59
Team competence and understanding	29.0 ± 4.2	13	35
Help and welcoming care	12.3 ± 1.9	4	15
Physical conditions of the service	8.2 ± 1.2	2	10

The data allowed identifying an overall change overview according to the factors and total score, as well as descriptive measures of the scores on the PCS among the university students who took part in the activities offered by ProACE, indicating 88.3% prevalence for change.

The results obtained in the subscales showed that the mean scores ranged between 2.25 (SD = 0.4) in the “Physical Activity and Health” dimension, 2.5 (SD 0.4) in the “Psychological Aspects and Sleep” dimension and 2.5 (SD = 0.4) in the “Relationships and Emotional Stability” dimension, with a range of 2.00–2.83 within a possible range of 1–3 (Table 4).

**TABLE 4:** Descriptive measures corresponding to the Perceived Change Scale scores in the university students who took part in the activities offered by ProACE.

Variables	*N*	Mean	Standard deviation
Physical activity and health	540	2.25 (2.00–2.63)	0.4
Psychological aspects and sleep	540	2.5 (2.00–2.83)	0.4
Relationships and emotional stability	540	2.5 (2.00–2.75)	0.4
*N*	540		

It was found that the bivariate associations obtained through simple linear regression for SATIS‐BR (with *p* < 0.5) indicated that the “Other” gender category, the “Indigenous” and “Brown” skin color/race categories, “fourth year of the program” and “presence of academic lag” were associated with lower mean scores on the scale, reflecting lower satisfaction. On the other hand, a positive result was observed for the campuses (notably B, followed by D) and for number of participations, showing that students with two participations reached higher scores when compared to the reference category (Table 5).

**TABLE 5:** Bivariate associations by simple linear regression for SATIS‐BR.

Variables	*b*	95% CI		*p*
Gender				
Other	−3.60	−6.79	−0.40	0.027
Trans woman	−2.63	−9.09	3.84	0.426
Cisgender woman	−0.35	−1.62	0.92	0.592
Cisgender man	0^a^			
Race/ethnicity				
Indigenous	−3.43	−5.82	−1.05	0.005
Black	−1.27	−3.32	0.77	0.222
Brown	−1.60	−3.18	−0.03	0.046
Asian	−2.90	−6.78	0.98	0.143
White	0^a^			
Campus				
D	2.82	0.80	4.84	0.006
C	−0.47	−1.92	0.98	0.527
B	5.82	3.59	8.04	0.000
A	0^a^			
Year of study				
Seventh	−2.18	−6.57	2.20	0.329
Sixth	−0.14	−2.53	2.24	0.907
Fifth	0.90	−1.82	3.62	0.515
Fourth	−3.82	−6.28	−1.36	0.002
Third	0.33	−1.73	2.40	0.751
Second	−1.19	−2.75	0.37	0.134
First	0^a^			
Academic lag				
Yes	−1.38	−2.62	−0.14	0.030
No	0^a^			
Number of participations				
2	6.33	1.46	11.19	0.011

^a^Reference category.

Presenting the bivariate associations obtained through simple linear regression for the overall PCS scale, Table 6 shows greater change perception among students at Campus B when compared to those from Campus A (reference). Regarding the interventions implemented, a positive association was observed between students who received psychological counseling and those who participated in Circle Dance. In contrast, lower mean scores on the scale were found among students who were provided assistance from a social worker and guidance on scholarships or housing assistance, as well as among those enrolled in third to seventh year of the program.

**TABLE 6:** Bivariate associations by simple normal linear regression for the overall PCS.

Variables	*b*	95% CI		*p*
Campus				
D	−0.07	−0.19	0.04	0.225
C	0.08	0.00	0.16	0.061
B	0.20	0.07	0.33	0.002
A	0^a^			
Year of study				
Seventh	−0.18	−0.42	0.07	0.163
Sixth	−0.16	−0.29	−0.03	0.019
Fifth	−0.16	−0.31	−0.01	0.041
Fourth	−0.23	−0.37	−0.09	0.001
Third	−0.14	−0.26	−0.03	0.016
Second	−0.03	−0.12	0.05	0.434
First	0^a^			
Intervention				
Welcoming care/yes	−0.02	−0.12	0.07	0.669
Welcoming care/no	0^a^			
Psychologist consultation/yes	0.22	0.15	0.30	0.000
Psychologist consultation/no	0^a^			
Information on assistance resources/yes	−0.15	−0.32	0.03	0.095
Information on assistance resources/no	0^a^			
Nursing consultation/yes	0.08	−0.08	0.23	0.323
Nursing consultation/no	0^a^			
Guidance on scholarship/yes	−0.22	−0.34	−0.09	0.001
Guidance on scholarship/no	0^a^			
Medical consultation/yes	−0.04	−0.17	0.09	0.543
Medical consultation/no	0^a^			
Social worker assistance/yes	−0.13	−0.24	−0.02	0.020
Social worker assistance/no	0^a^			
Guidance on housing assistance/yes	−0.44	−0.71	−0.17	0.001
Guidance on housing assistance/no	0^a^			
Circle Dance/yes	0.37	0.06	0.67	0.019
Circle Dance/no	0^a^			
Circle discussion/yes	−0.03	−0.31	0.24	0.803
Circle discussion/no	0^a^			
Other activities/yes	−0.04	−0.13	0.06	0.454
Other activities/no	0^a^			

^a^Reference category.

Regarding the multiple linear regression results for SATIS‐BR (Table 7), it was observed that the lowest satisfaction mean values remained associated with students who self‐identified as “Other” in the gender category and as “Indigenous” in terms of skin color/race. In contrast, participation in Circle Dance exerted the highest positive impact on the scale score, indicating higher satisfaction among the participants. Furthermore, students from Campuses B and D reached significantly higher satisfaction scores when compared to those at Campus A (reference group).

**TABLE 7:** Normal multiple linear regression for SATIS‐BR.

Variables	*b*	95% CI		*p*
Gender				
Other	−3.199	−6.291	−0.106	0.043
Trans woman	−4.114	−10.198	1.971	0.185
Cisgender woman	−1.025	−2.302	0.253	0.116
Cisgender man	0^a^			
Race/ethnicity				
Indigenous	−2.924	−5.453	−0.395	0.023
Black	−0.949	−2.907	1.009	0.342
Brown	−1.407	−2.928	0.115	0.070
Asian	−2.582	−6.284	1.121	0.172
White	0^a^			
Campus				
D	3.699	1.607	5.791	0.001
C	−1.040	−2.502	0.423	0.163
B	4.887	2.614	7.161	0.000
A	0^a^			
Intervention				
Welcoming care/yes	−5.726	−11.462	0.010	0.050
Welcoming care/no	0^a^			
Psychologist consultation/yes	0.278	−1.212	1.768	0.714
Psychologist consultation/no	0^a^			
Information on assistance resources/yes	1.315	−1.475	4.105	0.356
Information on assistance resources/no	0^a^			
Nursing consultation/yes	−0.449	−2.509	1.611	0.669
Nursing consultation/no	0^a^			
Guidance on scholarship/yes	−1.358	−5.983	3.267	0.565
Guidance on scholarship/no	0^a^			
Medical consultation/yes	6.984	1.638	12.330	0.010
Medical consultation/no	0^a^			
Social worker assistance/yes	−0.994	−6.046	4.058	0.700
Social worker assistance/no	0^a^			

^a^Reference category.

Presenting the multiple linear regression results for the overall PCS, Table 8 allows noticing a positive change perception in the “campus” and “activities” variables, particularly in consultations with a psychologist and in Circle Dance. With regard to the campuses, B showed the greatest perceived change in relation to the reference group. Negative variations were also observed in the variables related to academic year (particularly fourth year) and guidance on support services, which showed the lowest values for perceived changes.

**TABLE 8:** Normal multiple linear regression for the overall PCS.

Variables	*b*	95% CI		*p*
Campus				
D	−0.044	−0.161	0.074	0.465
C	0.047	−0.036	0.130	0.270
B	0.183	0.058	0.308	0.004
A	0^a^			
Year of study				
Seventh	−0.140	−0.391	0.112	0.276
Sixth	−0.103	−0.261	0.056	0.204
Fifth	−0.079	−0.245	0.088	0.355
Fourth	−0.158	−0.306	−0.009	0.037
Third	−0.092	−0.214	0.030	0.139
Second	−0.025	−0.117	0.068	0.601
First	0^a^			
Intervention				
Welcoming care/yes	0.040	−0.279	0.358	0.807
Welcoming care/no	0^a^			
Psychologist consultation/yes	0.185	0.098	0.273	0.000
Psychologist consultation/no	0^a^			
Information on assistance resources/yes	−0.043	−0.211	0.126	0.620
Information on assistance resources/no	0^a^			
Guidance on scholarship/yes	−0.088	−0.221	0.045	0.193
Guidance on scholarship/no	0^a^			
Social worker assistance/yes	−0.286	−0.548	−0.024	0.032
Social worker assistance/no	0^a^			
Guidance on housing assistance/yes	0.317	0.016	0.617	0.039
Guidance on housing assistance/no	0^a^			

^a^Reference category.

## 5. Discussion

Regarding the sociodemographic data found in this study, it was noticed that most of the participants were cisgender women (53.5%), white‐skinned (59.3%), aged between 21 and 24 years old (42%), and attending first year (41.9%).

Similar findings were reported in a study on the socioeconomic profile corresponding to undergraduate students at Brazilian Federal Higher Education Institutions (FHEIs), conducted in 2018 [18]. That research revealed a consistent increase in the female population within the university sector, with women representing 51.4% of the student population at FHEIs in 1996, reaching 54.6% in 2018. This phenomenon may be explained by the fact that women already constitute the majority at the high school level and, consequently, male students who complete or drop out of this level tend to enter the labor market at early ages [18].

As predicted, the proportion of students who took part in the survey and received some form of financial assistance or scholarship was 60.4%. This phenomenon can be attributed to the fact that the initiatives offered by ProACE are primarily aimed at students facing high vulnerability levels.

The university analyzed in this study participates in the National Student Assistance Program (Programa Nacional de Assistência Estudantil, PNAES), aimed at improving students’ retention conditions in federal public higher education through the provision of support benefits [19]. Nevertheless, it is worth noting that the percentage of students from families with monthly per capita incomes of “up to one and a half minimum wages” rose from 44.3% in 1996 to 66.2% in 2014, reaching 70.2% in 2018, the highest level in the historical series [18]. The research results revealed that 78% of the students did not live with their parents, indicating that they were not part of any family support system. This may have contributed to their emotional frailty, as they lacked the close support necessary to cope with their needs.

Leaving the family home proved to be a key characteristic among the most vulnerable students, and the transition to adulthood presented a new challenge. This period is characterized by a rupture and a learning process, oftentimes accompanied by an encounter with reality, idealized as perfect and ideal [20].

The program progression lag in relation to the expected year of study that was observed among 43% of the study participants can be attributed to several factors, including academic difficulties, excessive workload, personal problems, employment, changes in academic programs, lack of planning, health problems, lack of motivation, life changes, and disruptions resulting from university strikes.

The analysis of these factors associated with the assessed constructs indicated that the “health conditions,” “not living with parents,” “medication use,” and “academic life aspects” variables, as well as sociodemographic characteristics such as female gender and low income, are potential risk factors for psychological distress among students [21].

It is evident from the data presented that the challenge of ensuring students’ entry into university and their retention in an environment characterized by constant adaptation requires developing actions and programs that offer support to this population group.

Consequently, transdisciplinary projects that seek network partnerships both inside and outside the university are potential mechanisms for promoting well‐being.

Established by GM/MS Administrative Order No. 3088/2011, the Psychosocial Care Network (Rede de Atenção Psicossocial, RAPS) aims at coordinating health services throughout the region and at improving care quality through initial assessments, ongoing follow‐ups, and emergency care [22].

The university services in question value respect and dignity and foster a partnership with constant dialog and reciprocal referrals in the health and student assistance areas, both internal and external to the university. This fact can be observed in several SATIS scale items.

In this way, articulated with an internal policy that promotes student‐centered mental health, joint action by professionals and departments can help make their university experience more positive, reducing illness risks and promoting well‐being [5].

According to the activities listed, the most frequently observed was consultation with a psychologist, in 28.7% of the individual activities recorded.

In turn, group activities have been expanding across all four campuses, reinforcing the psychosocial care model that includes integrative and complementary practices (ICPs), given their low cost and potential to promote health [23].

However, it is clear that this type of activity requires support not only from its organizers but also from the community at large. The low student participation highlights the need to reevaluate strategies for engaging students in collective practices. As it focuses on stress management, promotion of empathy, mental health, and academic performance, mindfulness stands out among the initiatives analyzed [24].

By also reducing perceived stress and increasing overall perceived self‐efficacy, it may contribute to managing challenging situations in the academic context, during training and in the future professional practice [25].

Regarding the activities related to welcoming, both individual and collective, it is observed that they offer students greater likelihood of establishing satisfactory interpersonal relationships, affective bonds, and group identification. In turn, this exerts a favorable impact on adaptation and belonging processes [26, 27].

The purpose of welcoming care is to receive each person in a receptive and attentive manner, identifying risks, problems, and vulnerabilities with the intention of assisting in solving problems through responsible and effective support [28].

Interventions of this nature have shown to be significant tools for developing professional and interpersonal skills [6]; in addition, devising broad and cross‐sectional welcoming strategies has shown to be of significant benefit for universities [27].

Consequently, these services should adopt a student‐centered approach, ensuring a supportive and easily accessible environment that fosters a confidential, inclusive, and safe atmosphere where students feel respected and valued [29].

It has been shown that welcoming care is essential for maintaining good mental health, as evidenced during the academic period [3].

The cultural, leisure, physical, and sporting activities offered contribute to promoting well‐being and act as protective factors for mental health [30]. These activities are encouraged across all four campuses as beneficial factors for good mental health, with particular emphasis on Circle Dance, which was observed in one of the four campuses.

Welcoming environments with health‐promoting practices such as leisure, physical activity, and sports can enhance the students’ performance by providing enjoyment, highlighting that well‐being results from positive interactions between individuals and their environment, occupation, and performance [5].

According to Alcântara, Simon, Guimarães [31], the extension and cultural activities found in universities are capable of influencing and contributing much more than the university itself can propose as an activity, through the perspective of promoting health and mental health.

Given that universities constitute institutional, political, social, and cultural spaces capable of engaging with the surrounding society, fostering knowledge production, and fulfilling their social mission, concern extends beyond merely training technical professionals to also include preparing individuals to take part in citizenship construction [32].

Furthermore, health monitoring through the network should be encouraged, as comorbidities associated with mental health problems among students are more severe with greater extent of each comorbidity [33].

In this context of providing care through teams to students experiencing psychological distress, a recurrent complaint is noticed: scarce participation from the university community itself in the actions offered by the departments. This reflects the students’ reluctance to seek support, even when services are in fact available, a characteristic that may be influenced by customs, beliefs, and social stigmas surrounding mental health [29, 34].

The entire university community should reinforce the idea that, from the students’ perspective, seeking help is not a sign of weakness, but rather a sign of strength and confidence [29].

An alternative identified in this study to improve adherence to activities would be the involvement of other sectors and university administrative offices in student care, reducing bureaucratic barriers to referrals within the university, promoting a healthier overall educational environment, and ensuring that such activities become fixed components of the academic calendar.

When analyzing the students’ perceptions regarding change, 88% indicated that they have been feeling better since the activities and services designed to address mental distress were initiated. This percentage was considered favorable, as the activities offered were perceived as beneficial and potentially contributed to improvements in relevant aspects of their academic, personal, and emotional life.

The perception that positive changes have taken place in their lives as a result of the treatments undergone in the services is important because it influences the relationship between objective treatment outcomes and user satisfaction degrees [8].

When comparing the data to those from other studies, it was found that there are yet no surveys assessing university students’ change perception using PCS in the mental health field. The studies involving students that were identified analyzed specific activities leading to an improvement in overall well‐being, but did not use this scale.

For example, the provision of ICPs at another Brazilian Federal university proved to be pioneering in its transdisciplinary and interprofessional approach to preventing intensification of anxiety disorders, thus reducing the need for medications [23]. These studies reveal different variables of possible actions for coping with psychological distress in university students.

Although the SATIS‐BR and overall PCS were originally developed and validated in the context of clinical and mental health services, their application in this study within a university health promotion setting is grounded in the multidimensional nature of biopsychosocial well‐being. ProACE’s student assistance programs serve as preventive and psychosocial support interventions. Therefore, the construct validity of these instruments is supported by the premise that satisfaction with the service and personal change perceptions are cross‐cutting constructs, equally applicable for assessing the perceived effectiveness of retention and care policies outside the strictly outpatient setting.

In this regard, having used scales made it possible to analyze subjective aspects of the students perceptions regarding the changes that took place following interventions aimed at promoting mental health, thereby broadening our understanding of the associations between these actions. Furthermore, some studies have highlighted the importance of instruments that consider the students’ own perspectives in evaluating psychosocial support programs and services, reinforcing suitability of the construct for this context [9].

In addition, content validity supports the suitability of applying these scales, as the instruments’ items remain representative of the conceptual domain researched. In the university context, aspects related to emotional well‐being, interpersonal relationships, the ability to cope with difficulties, and performance in daily activities closely correspond to the domains traditionally assessed in mental health services. Thus, the instruments’ content retains its theoretical and practical relevance for assessing the changes perceived by students [35].

The high mean scores for overall satisfaction and perceived change indicate that the care practices provided were well received by the students and positively associated with reports of improvements in their well‐being. Convergence of these indicators reinforces the relevance of institutional initiatives, as the participants stated endorsing the services received and held favorable perceptions regarding the impacts on their academic paths. These results have practical relevance for strengthening retention and mental health policies in higher education, as they indicate that support and care services meet the students’ psychosocial support needs, subject to the cross‐sectional nature of the study design.

Furthermore, the findings of this study should be interpreted in the light of selection and referral biases, which are inherent to observational study designs applied to student assistance services. It was observed that variables indicating greater structural vulnerability and demand for socioeconomic support such as need for housing assistance or services from a social worker remained associated with lower scores on the assessment scales. This inverse relationship should not be linearly interpreted as ineffectiveness or dissatisfaction with the services provided. On the contrary, this pattern reflects referral bias, whereby students who seek out these programs or are referred to them are already in greater social vulnerability, acute distress, or academic overload situations. Consequently, their needs are more complex and profound, turning change and satisfaction perceptions into a gradual and challenging process, which reinforces the need for continuous and integrated institutional support targeted at this population segment.

## 6. Limitations

The cross‐sectional design of this study constitutes a limitation, as data collection took place at a single point in time after the interventions. Although the research was able to assess user satisfaction and change perceptions, the absence of data prior to initiating the activities precludes assessing the participants’ baseline equivalence. Consequently, this limitation restricts the ability to establish definitive causal relationships, making it impossible to confirm the strict efficacy of the interventions or to exclusively attribute the perceived improvements in access to ProACE services.

The participants were already using the services and chose to take part in the survey, introducing selection bias. As a result, the sample may have predominantly consisted of students who were more motivated to evaluate the services or of those who had specific experiences, limiting representativeness of the results in relation to the overall group of students who resorted to the services.

Both satisfaction and perceived change measure users’ subjective experiences, which implies inherent limitations to this type of measurement. The literature indicates that answers on such scales can be influenced by social desirability and by the respondents’ tendency to provide socially acceptable or favorable responses to the service, as well as by memory biases, especially when assessing changes over time, as in the case of PCS. Thus, although they are valid and useful instruments for monitoring quality and perceived outcomes, their results should be interpreted with caution and, whenever possible, triangulated with other data sources (clinical, observational, or institutional) for a more robust assessment of mental health services, as the scales were used in a university setting.

In the context of future studies, it is recommended to consider the possibility of including open‐ended questions in addition to a closed questionnaire. This approach would provide a platform for students to articulate their perspectives on the activities/actions carried out, thus easing the interpretation of subjective issues. This recommendation stems from the remark that the current study only incorporated a single qualitative question, which may have limited the exploration scope for subjective dimensions.

The form did not have a specific completion deadline and was submitted only once, with participation in some activity or service offered as sole criterion.

## 7. Implications for Research and Practice

When comparing the data to those form other studies, it was noticed that no survey has yet assessed perceptions regarding change and satisfaction with services using the PCS and the SATIS instrument in the mental health area with university students. The studies found analyzed specific activities that improved overall health but did not use these scales.

Therefore, this article might exert a positive impact on the clinical practice, increasing awareness of mental health issues among university students and strengthening student‐centered care. Furthermore, it reinforces the fundamental role of nursing in promoting mental health, implementing preventive and interventional strategies, and advocating for public policies that improve community well‐being and strengthen the health system.

## 8. Recommendations

The satisfaction and perceived change constructs proved important in the analysis of the mental health service offered, being relevant in relation to the psychological distress observed in university students.

The hypothesis presented in this study was confirmed, revealing a statistically significant association between the treatments received and the changes perceived by the students. This finding suggests that the initiatives implemented by the university have played a significant role in addressing psychological distress, fostering positive change perceptions, and contributing to the students’ well‐being.

It was observed that the students were highly satisfied with the departments, staff, and actions offered by the university, as most of the participants reported a sense of positive change after attending such activities.

Promoting activity initiatives within the university to improve dissemination of projects both within the academic environment and outside the institution fosters greater student engagement in such activities and may exert positive impacts on mental health. When students become actively involved, they tend to feel more connected and motivated, reducing feelings of isolation and stress while inspiring other peers to participate, thereby creating a more collaborative and positive environment.

Implementing activities becomes important to encourage university students to seek help, including measures that promote stigma‐free and enjoyable environments, reduce psychological distress, and encourage using adaptive coping strategies.

Therefore, institutional efforts should be continuously encouraged, seeking partnerships and theoretical foundations both inside and outside the university, combined with the interprofessional work of health and student assistance teams, so that they can continue to contribute to the students’ experiences at the university, ensuring that they are permeated by successful experiences, reducing risk factors for illness and contributing to mental health.

## Author Contributions

Study design, data collection, data analysis, and final approval of the manuscript: Tatiane Carolina Martins Machado Rodrigues and Guilherme Correa Barbosa.

## Funding

No funding was received for this manuscript.

## Disclosure

All authors read and approved the final manuscript.

## Ethics Statement

The study was approved by the Research Ethics Committee of the Botucatu Medical School (Faculdadde de Medicina de Botucatu, FMB) belonging to the São Paulo State University (UNESP) (Protocol No. 5.766.479/2022 [CAAE: 64343022.4.0000.5411]), following Brazilian National Health Council Resolution No. 466/2012. All participants provided their written informed consent and were advised about their right to withdraw at any moment. In order to ensure confidentiality, the participants’ names were anonymized and their personal data were stored in a password‐protected database.

## Consent

Please see the Ethics Statement.

## Conflicts of Interest

The authors declare no conflicts of interest.

## Data Availability

The data that support the findings of this study are available from the corresponding author upon reasonable request.

## References

[1] Mango E. J. , Campos C. M. S. , Soares C. B. , and Trapé C. A. , Manifestations of Psychological Distress in Students from Different Social Groups at the University of São Paulo, Revista da Escola de Enfermagem da USP. (2024) 58, 10.1590/1980-220x-reeusp-2024-0231en. PMC11849675 39992180

[2] Illingworth P. , Culturally Sensitive, Localized, Universal Mental Health Care, Journal of Psychiatric and Mental Health Nursing. (2021) 28, no. 6, 941–942, 10.1111/jpm.12774. 34036685

[3] Medeiros L. R. , Rodrigues K. C. C. , Queiroz A. M. , Florencio R. M. S. , Silva A. F. , and Borges A. M. , Cartografia Dos Serviços De Acolhimento Ao Acadêmico Em Sofrimento Psíquico nas Universidades Públicas Brasileiras, Cogitare Enfermagem. (2022) 27, 10.5380/ce.v27i0.75756.

[4] Mota A. A. S. , Pimentel S. M. , and Mota M. R. S. , Expressões De Sofrimento Psíquico De Estudantes Da Universidade Federal do Tocantins, Educação e Pesquisa. (2023) 49, 10.1590/s1678-4634202349254990.

[5] Rodrigues D. S. and Santos J. E. , Cuidado Centrado En El Estudiante (CCE): Propuesta De Intervención Para La Promoción De La Salud Entre Los Universitarios Brasileños, Revista Ocupación Humana. (2025) 25, no. 1, 47–59, 10.25214/25907816.1792.

[6] Ramos F. P. , Andrade A. L. , Jardim A. P. , Ramalhete J. N. L. , Pirola G. P. , and Egert C. , Intervenções Psicológicas Com Universitários em Serviços De Apoio Ao Estudante, Revista Brasileira de Orientação Profissional. (2018) 19, no. 2, 221–232.

[7] World Health Organization , Global Status Report on Alcohol and Health 2018, 2019, https://www.who.int/publications/i/item/9789241565639.

[8] Bandeira M. B. , Andrade M. C. R. , Costa C. S. , and Silva M. A. , Percepção Dos Pacientes Sobre O Tratamento em Serviços De Saúde Mental: Validação Da Escala De Mudança Percebida, Psicologia: Reflexão e Crítica. (2011) 24, no. 2, 236–244, 10.1590/s0102-79722011000200004.

[9] Martins F. , A Satisfação Acadêmica: Construção De Uma Escala, Em Anais do 4 Congresso Galaico-Português De Psicopedagogia, 1998. (1998) Universidade do Minho, Braga, Portugal.

[10] Donabedian A. , Basic Approaches to Assessment: Structure, Process and Outcome, Heath Administration Press. (1980) 1, 77–125.

[11] Cuschieri S. , The STROBE Guidelines, Saudi Journal of Anaesthesia. (2019) 13, no. Suppl 1, S31–S34, 10.4103/sja.SJA_543_18. PMC6398292 30930717

[12] Brasíl Resolução no 466 , Aprova Diretrizes e Normas Regulamentadoras de Pesquisas Envolvendo Seres Humanos [Internet], 2012, Ministério da Saúde, Brasília, https://conselho.saude.gov.br/resolucoes/2012/Reso466.pdf.

[13] World Health Organization , WHO-SATIS Consumer’s and Caregivers’ Satisfaction with Mental Health Services a Multisite Study, Mental Health World Organization. (1996) .

[14] Bandeira M. and Silva M. A. , Escala De Satisfação Dos Pacientes Com Os Serviços De Saúde Mental (SATIS-BR): Estudo De Validação, Jornal Brasileiro de Psiquiatria. (2012) 61, no. 3, 124–132, 10.1590/s0047-20852012000300002.

[15] Bandeira M. , Pitta A. M. F. , and Mercier C. , Escala De Avaliação Da Satisfação dos Usuários em Serviços De Saúde Mental: SATIS-BR, Jornal Brasileiro de Psiquiatria. (2000) 49, no. 8, 293–300.

[16] Mercier L. , Landry M. , Corbière M. , and Perreault M. , Roberts A. R. and Yeager (Orgs) K. R. , Measuring Clients Perception as Outcome Measurement, Evidence-Based Practice Manual: Research an Outcome Measures in Health and Human, 2004, Oxford University Press, 904–909.

[17] Bandeira M. , Calzavara M. G. P. , Costa C. S. , and Cesari L. , Avaliação De Serviços De Saúde Mental: Adaptação Transcultural De Uma Medida Da Percepção Dos Usuários Sobre Os Resultados do Tratamento, Jornal Brasileiro de Psiquiatria. (2009) 58, no. 2, 107–114, 10.1590/s0047-20852009000200007.

[18] Associação Nacional dos Dirigentes das Instituições Federais de Ensino Superior , V Pesquisa Nacional De Perfil Socioeconômico E Cultural Dos (As) Graduandos (As) Das IFES: 2018, 2018, http://www.fonaprace.andifes.org.br/site/wp-content/uploads/2019/06/V-Pesquisa-do-Perfil-Socioecono%CC%82mico-dos-Estudantes-de-Graduac%CC%A7a%CC%83o-das-U.pd.

[19] Brasil Decreto no 7.234 , Dispõe sobre o Programa Nacional de Assistência Estudantil-PNAES [Internet], 2010, Presidõncia da República, Brasília, https://www.planalto.gov.br/ccivil_03/_ato2007Dispon%edvelem:2010/2010/decreto/d7234.htm#:~:text=Decreto%20n%C2%BA%207234%26text=DECRETO%20N%C2%BA%207.234%2C%20DE%2019,que%20lhe%20confere%20o%20art.

[20] Cunha E. C. M. , Souza A. B. R. , and Novaes V. R. , Sofrimento Psíquico De Estudantes No Ensino Superior: Uma Revisão Sistemática Da Literatura, RECIMA21. (2022) 3, no. 5, https://recima21.com.br/index.php/recima21/article/view/1460/1122, 10.47820/recima21.v3i5.1460.

[21] Graner K. M. and Cerqueira A. T. A. R. , Revisão Integrativa: Sofrimento Psíquico em Estudantes Universitários E Fatores Associados, Ciência & Saúde Coletiva. (2019) 24, no. 4, 1327–1346, 10.1590/1413-81232018244.09692017. 31066836

[22] Brasil Ministério da Saúde , Portaria de Consolidação RAPS 3/2017, 2025, Diário Oficial da, https://bvsms.saude.gov.br/bvs/saudelegis/gm/2017/prc0003_03_10_2017ARQUIVO.html.

[23] Belasco I. C. , Passinho R. S. , and Vieira V. A. , Práticas Integrativas E Complementares Na Saúde Mental do Estudante Universitário, Arquivos Brasileiros de Psicologia. (2019) 71, no. 1, 103–111, 10.36482/1809-5267.ARBP2019v71i1p.103-111.

[24] Silveira L. L. , Fabrizzi H. E. , Hamilko H. C. C. , Stefanello S. , and Santos D. V. D. , Os Efeitos Do Mindfulness Na Percepção Dos Estudantes De Medicina De Uma Universidade Brasileira, Revista Brasileira de Educação Médica. (2021) 45, no. 2, 10.1590/1981-5271v45.2-20200149.

[25] Azevedo M. L. and Menezes C. B. , Efeitos Do Programa Terapia Cognitiva Baseada em Mindfulness Sobre Estresse, Autoeficácia E Mindfulness em Universitários, SMAD, Revista Eletrônica Saúde Mental Álcool e Drogas. (2020) 16, no. 3, 44–54, 10.11606/issn.1806-6976.smad.2020.165513.

[26] Soares A. B. , Francischetto V. , Dutra B. M. et al., O Impacto Das Expectativas Na Adaptação Acadêmica Dos Estudantes No Ensino Superior, Psico-USF. (2014) 19, no. 1, 49–60, 10.1590/s1413-82712014000100006.

[27] Albanaes P. , Bardagi M. P. , Luca G. G. , and Girelli S. , Do Trote À Mentoria: Levantamento Das Possibilidades De Acolhimento Ao Estudante Universitário, Revista Brasileira de Orientação Profissional. (2014) 15, no. 2, 143–152, https://pepsic.bvsalud.org/scielo.php?script=sci_arttext%26pid=S1679-33902014000200005.

[28] Kuse E. A. , Taschetto L. , and Cembranel P. , O Cuidado Na Saúde Mental: Importância do Acolhimento Na Unidade De Saúde, Revista Espaço para a Saúde. (2022) 23, e874–10, 10.22421/1517-7130/es.2022v23.e874.

[29] Dagani J. , Buizza C. , Ferrari C. , and Ghilardi A. , The Role of Psychological Distress, Stigma and Coping Strategies on help-seeking Intentions in a Sample of Italian College Students, BMC Psychology. (2023) 11, no. 1, 10.1186/s40359-023-01171-w. PMC10243082 37280661

[30] Pondé M. P. and Caroso C. , Lazer Como Fator De Proteção Da Saúde Mental, Revista de Ciencias Medicas. (2003) 12, no. 2, 163–172, https://seer.sis.puc-campinas.edu.br/cienciasmedicas/article/view/1268/1242.

[31] Alcântara L. D. F. de , Simon C. R. , and Guimarães R. B. , Saúde Mental E O Contexto Universitário:: Uma Leitura Interseccional Das Juventudes Na FCT-UNESP, de fevereiro de 2024. (2024) 4, no. 1, 03–21, https://www.geoconexoesonline.com/revista/article/view/152, 10.53528/geoconexes.v4i1.152.

[32] Rodrigues T. C. M. M. and Jesus S. F. C. , Cotrim F. S. , Duval H. C. , and Peres A. M. P. , Calourada Solidária do Campus Lagoa do Sino: Combate Ao Mosquito Aedes aegypti, (Orgs), Dez Anos do Campus Lagoa do Sino Da Ufscar: Memórias E Experiências, 2025, EdUFSCar, 387–392.

[33] Berman A. H. , Topooco N. , Lindfors P. et al., Transdiagnostic and Tailored Internet Intervention to Improve Mental Health Among University Students: Research Protocol for a Randomized Controlled Trial, Trials. (2024) 25, no. 1, 10.1186/s13063-024-07986-1. PMC10908025 38429834

[34] Beroíza-Valenzuela F. , The Challenges of Mental Health in Chilean University Students, Frontiers in Public Health. (2024) 12, 10.3389/fpubh.2024.1297402. PMC10963499 38532969

[35] Messick S. , Validity of Psychological Assessment: Validation of Inferences from Persons’ Responses and Performances as Scientific Inquiry into Score Meaning, American Psychologist. (1995) 50, no. 9, 741–749, 10.1037/0003-066x.50.9.741.

